# Health Food for the Multitude

**Published:** 1878-07

**Authors:** 


It was a great comfort to us to be able to announce, as we did las[illegible] autumn, that a leading first-class hotel in New York—the STURTEVAN[illegible] HOUSE—had added several of the delicate products of the Health Food Co. to its bill of fare. The fact indicated that the great reforms in th[illegible] modes of preparing well-known food substances, had penetrated othe[illegible] minds besides those of the doctors and scientists, and that the more enterprising of those who cater for the public palate had determined tha[illegible] full compensation might be depended upon for those who should seek t[illegible] intelligently supply the new demand.

The proprietors of the STURTEVANT—the Messrs. LELAND—assure us that their conclusions were correct, and that the results have been very gratifying. The public have indicated their approval of the addition o[illegible] the HEALTH FOODS to the vast schedule of substantials and delicacies always provided, by warmer encomiums, which have led to increased patronage. Thus the Sturtevant has been full to the brim, while othe[illegible] hotels, of good repute, have suffered for lack of patronage in this perio[illegible] of depression.

Our object in writing these lines is to call the attention of the publi[illegible] to a similar new departure on the part of another large hotel and resta[illegible]rant in the city of New York. The latest establishment to provide th[illegible] products of the Health Food Company for guests, is the large hotel extending from 195 to 199 Washington St., in this city, an institution long established, of very high repute, and which provides food for a large[illegible] number of persons daily than any other house in the world. In this vast establishment over 2,000,000 persons dine each year; and within the walls of the great edifice 100,000 men annually find luxurious lod[illegible]ment. The house is probably the best ventilated building on the co[illegible]nent, a complete system of air supply and purification having been [illegible]vided for it by Mr. C. Hartwell. The proprietors are Messrs. Henry S[illegible] and Thomas R. McNell, and the institution is known as "SMITH & [illegible]NELL'S HOTEL AND RESTAURANT." Visitors to their spacious dinin[illegible] rooms, now read in all directions, "HEALTH FOODS from the HEALT[illegible] FOOD COMPANY, 74 Fourth Avenue." The firm, having made a carefu[illegible] study of the laws of health, have introduced the new life-giving foods t[illegible] the 6,000 or 8,000 daily patrons of their house; and it is their convicti[illegible] that the HEALTH FOOD COMPANY of New York are doing a noble work f[illegible] humanity, a work in the benefits of which all mankind should be allowe[illegible] to participate. Everybody prospers who supplies these perfect foods.

---

DR. TRALL'S CRACKERS.—Dr. R. T. Trall, the noted hygienic writer, died some months sinc[illegible] but his works live after him. Our advertising pages state that his celebrated crackers are still ma[illegible] by Chas. H. Hoyt & Son, 436 Greenwich Street. The Messrs. Hoyt are thoroughly reliable bake[illegible] and their goods have no superior anywhere.

---

A CONCENTRATED FOOD.—The HEALTH FOOD COMPANY'S latest and greatest achievemen[illegible] found, we think, in thei EXTRACT OF GLUTEN AND BARLEY. We have been watching its effects i[illegible] two cases of tuberculosis, in one of which the temperature had stood at 105° for eleven days, indicating enormous destruction of tissue. The result was exceedingly gratifying, the heat dropping t[illegible] 100° in four days. In both cases the symptoms and condition have greatly improved. We long ag[illegible] gave up cod-liver oil in the treatment of lung troubles, on account of its power to derange digestion: and we believe we have found a perfect substitute for the nauseous oil, in this delicious and high[illegible] nutritious extract of Gluten and Barley. Our friends who want some valuable hygienic literatu[illegible] should ask the HEALTH FOOD COMPANY for pamphlets.

